# Antivirulence activities of retinoic acids against *Staphylococcus aureus*

**DOI:** 10.3389/fmicb.2023.1224085

**Published:** 2023-09-13

**Authors:** Inji Park, Jin-Hyung Lee, Jin Yeul Ma, Yulong Tan, Jintae Lee

**Affiliations:** ^1^School of Chemical Engineering, Yeungnam University, Gyeongsan, Republic of Korea; ^2^Korea Institute of Oriental Medicine, Daegu, Republic of Korea; ^3^Special Food Research Institute, Qingdao Agricultural University, Qingdao, China

**Keywords:** antivirulence, biofilm, hemolysis, retinoic acid, *Staphylococcus aureus*, vitamin A_1_

## Abstract

Multidrug-resistant bacteria such as *Staphylococcus aureus* constitute a global health problem. Gram-positive *S. aureus* secretes various toxins associated with its pathogenesis, and its biofilm formation plays an important role in antibiotic tolerance and virulence. Hence, we investigated if the metabolites of vitamin A_1_ might diminish *S. aureus* biofilm formation and toxin production. Of the three retinoic acids examined, 13-*cis*-retinoic acid at 10 μg/mL significantly decreased *S. aureus* biofilm formation without affecting its planktonic cell growth (MIC >400 μg/mL) and also inhibited biofilm formation by *Staphylococcus epidermidis* (MIC >400 μg/mL), but less affected biofilm formation by a uropathogenic *Escherichia coli* strain, a *Vibrio* strain, or a fungal *Candida* strain. Notably, 13-*cis*-retinoic acid and all-*trans*-retinoic acid significantly inhibited the hemolytic activity and staphyloxanthin production by *S. aureus*. Furthermore, transcriptional analysis disclosed that 13-*cis*-retinoic acid repressed the expressions of virulence- and biofilm-related genes, such as the two-component *arlRS* system, α-hemolysin *hla*, nuclease (*nuc1* and *nuc2*), and *psmα* (phenol soluble modulins α) in *S. aureus*. In addition, plant and nematode toxicity assays showed that 13-*cis*-retinoic acid was only mildly toxic at concentrations many folds higher than its effective antibiofilm concentrations. These findings suggest that metabolites of vitamin A_1_, particularly 13-*cis*-retinoic acid, might be useful for suppressing biofilm formation and the virulence characteristics of *S. aureus*.

## 1. Introduction

Infections caused by drug-resistant bacteria are increasing globally, but the rate of novel antibiotic discovery has declined continuously over past decades. Accordingly, other treatment strategies, such as antitoxin and antibiofilm-based approaches, are being investigated to cope with drug-resistant microbes. Unlike antimicrobial agents, antivirulence compounds are required to reduce the virulence characteristics of microbes without negatively affecting cell growth because microbial killing is associated with a higher risk of developing drug resistance (Dickey et al., 2017).

*Staphylococcus aureus* is a main cause of community-acquired and nosocomial infections due to its multidrug resistance. This bacterium produces various virulence factors, such as hemolysin, enterotoxins, and immune evasive staphyloxanthin, and causes diverse life-threatening infections, including bacteremia, pulmonary infections, gastroenteritis, toxic shock syndrome, and skin infections (Tong et al., 2015). *S. aureus* readily forms chronic biofilms on host cells and medical devices and implants. Moreover, this ability to form biofilms plays critical roles in antibiotic tolerance and virulence and significantly increases morbidity and mortality rates, particularly when associated with indwelling medical devices (Moormeier and Bayles, 2017). Hence, inhibiting virulence factor production and biofilm formation (Winkelströter et al., 2014; Park et al., 2022) offer alternative means of fighting recalcitrant *S. aureus* infections.

Although the antibiofilm potential of vitamins has long been suggested, antibiofilm activities have been attributed to relatively few, such as vitamin B_12_ against *Pseudomonas aeruginosa* (Lee et al., 2012), vitamin C against *Escherichia coli* (Shivaprasad et al., 2021), and *Klebsiella pneumoniae* (Xu et al., 2022), and vitamin D against *S. aureus* (Vergalito et al., 2018). However, no study has yet investigated the antibiofilm characteristics of vitamin metabolites.

In this study, we sought to identify a compound that inhibits biofilm formation and toxin production of *S. aureus* without killing the bacterium. Three metabolites of vitamin A_1_ (all-*trans*-retinoic acid, 9-*cis*-retinoic acid, and 13-*cis*-retinoic acid) were initially investigated for their antibiofilm activity against *S. aureus*. The most active, 13-*cis*-retinoic acid, was further investigated for the activity against three other *Staphylococcus* strains, a *Staphylococcus epidermidis* strain, an uropathogenic *Escherichia coli* strain, a *Vibrio parahaemolyticus* strain, and a *Candida* strain. Live imaging microscopy, scanning electron microscopy, qRT-PCR, and hemolysis and lipase activities were used to investigate how 13-*cis*-retinoic acid affects biofilm formation and toxin production of *S. aureus*. In addition, the toxicity of 13-*cis*-retinoic acid was investigated using nematode *Caenorhabditis elegans* and plant *Brassica rapa* models, and ADME simulation was performed.

## 2. Materials and methods

### 2.1. Bacterial strains, culture media, chemicals, and growth analysis

Two methicillin-sensitive *S. aureus* strains (MSSA; ATCC 6538 and ATCC 25923) and two methicillin-resistant *S. aureus* strains (MRSA 33591 and MW2), an *S. epidermidis* strain (ATCC 14990), a fungal *Candida albicans* DAY185 strain, a uropathogenic *E. coli* O6:H1 strain CFT073 (ATCC 700928), and a *Vibrio parahaemolyticus* strain ATCC 17802 were used. Cultures of MSSA ATCC 6538, ATCC 25923, and *S. epidermidis* strains were performed in Luria-Bertani (LB) broth, and MRSA ATCC 33591 and MW2 strains were cultivated in LB medium containing 0.2% glucose at 37°C and 30°C. *Candida albicans* DAY185 was cultured in potato dextrose broth (PDB). UPEC and *V. parahaemolyticus* were cultured in nutrient broth (NB) and LB supplemented with 3% (w/v) NaCl (mLB) at 37°C, respectively. All-*trans*-retinoic acid, 9-*cis*-retinoic acid, 13-*cis*-retinoic acid, and crystal violet were obtained from Sigma-Aldrich (St. Louis, MO, United States). Dimethyl sulfoxide (DMSO) was used to dissolve retinoic acids, and DMSO (0.1% v/v) was used as a control and it did not affect cell growth or biofilm formation. For planktonic cell growth assay, colony-forming units (CFU) were determined after incubating *S. aureus* cells in 96-well plates in LB medium with or without 13-*cis*-retinoic acid for 24 h.

### 2.2. Crystal-violet biofilm assay

A crystal violet staining assay was conducted using 96-well plates, as previously reported (Lee et al., 2021). *S. aureus* cells (~10^7^ CFU/mL) were inoculated into LB medium and retinoic acids were added at 0, 2, 5, 10, 20, 50, or 100 μg/mL to the wells of 96-well plates and cultivated for 24 h at 37°C without agitation. Biofilm formation was measured by discarding planktonic cells and washing the plates three times with distilled water. Biofilm cells were then stained with 0.1% crystal violet (300 μL) for 20 min and washed three times with water to remove crystal violet. Crystal violet stained cells were then extracted with 95% ethanol (300 μL) by shaking vigorously. Absorbances were measured at 570 nm (OD_570_) using a Multiskan plate reader (Thermo Fisher Scientific, Waltham, MA, United States). Biofilm formation results are obtained from three independent cultures of six replicate wells.

### 2.3. Biofilm observations by microscopies

After forming *S. aureus* biofilms in 96-well plates in the presence or absence of 13-*cis*-retinoic acid (0, 2, 5, or 10 μg/mL) for 24 h at 37°C, planktonic cells were removed by washing three times with distilled water, and live biofilm cells were observed by the iRiS™ Digital Cell Imaging System (Logos Biosystems, Anyang, Korea). Color-coded 3D biofilm images were generated using ImageJ.^1^

Also, SEM was used to observe biofilm reduction by 13-*cis*-retinoic acid, as previously reported (Park et al., 2022). *S. aureus* ATCC 6538 cells (~10^7^ CFU/mL) were inoculated into 1 mL of fresh LB medium with or without 13-*cis*-retinoic acid (0, 2, 5, or 10 μg/mL) in a 96-well plate. A piece of nylon membrane (~ 0.16 cm^2^) was placed in each well, and *S. aureus* cells were cultured for 24 h at 37°C without agitation. Biofilms developed on the membrane were then fixed with a glutaraldehyde (2.5%) and formaldehyde (2%) for 24 h, post-fixed with OsO_4_ (1%), and dehydrated with ethanol and isoamyl acetate (99%). After drying biofilms using critical-point dryer (HCP-2, Hitachi, Tokyo, Japan), biofilm cells were coated with Precision Etching Coating System (Gatan, Inc., Pleasanton, United States) and observed under a field emission scanning electron microscope S-4800 (Hitachi, Tokyo, Japan).

### 2.4. Hemolytic activity assay

The hemolysis of sheep blood cells (MBcell, Seoul, Korea) was investigated as described previously (Kim et al., 2022a). *S. aureus* ATCC 6538 cells (~2 × 10^7^ CFU/mL) were diluted in 2 mL of fresh LB medium, cultivated with retinoic acids (0, 0.5, 1, 2, 5, or 10 μg/mL) for 24 h with 250 rpm shaking. Fresh sheep blood cells were collected by centrifugation at 3,000 × g for 2 min, the red blood cells were then cleaned three times with PBS and resuspended gently in PBS buffer (3.3%). *S. aureus* cell culture (100 μL) was then added to 1 mL of red blood cells and incubated for 4 h at 37°C with shaking at 250 rpm. The mixtures were centrifugated at 16,600 × g for 10 min, and the absorbances of supernatants were measured at 543 nm.

### 2.5. Staphyloxanthin production assay

*Staphylococcus aureus* ATCC 6538 cells (~2 × 10^7^ CFU/mL) were inoculated into LB medium (2 mL) in 14-mL tubes and incubated for 24 h at 37°C with 13-*cis*-retinoic acid (0, 10, 20, 50, or 100 μg/mL) with shaking at 250 rpm. Staphyloxanthin levels were assessed optically, as previously described (De Souza Feitosa Lima et al., 2019; Kim et al., 2022a).

### 2.6. Extracellular lipase production assay

To quantify the effect of 13-*cis-*retinoic acid on extracellular lipase production, *S. aureus* ATCC 6538 cells (~2×10^7^ CFU/mL) were inoculated into LB medium (2 mL) in 14-mL tubes and incubated for 20 h at 37°C with 250 rpm shaking with or without 13-*cis*-retinoic acid (0, 2, 5, 10, 20, or 50 μg/mL), as previously reported (Lee et al., 2022). Briefly, culture supernatants (0.1 mL) were mixed with 0.9 mL of substrate solution (10% of buffer A with 3 mg/mL of p-nitrophenyl palmitate in isopropyl alcohol and 90% of buffer B with 1 mg/mL of gum arabic and 2 mg/mL sodium deoxycholate in 50 mM Na_2_PO_4_ buffer and then heated at 40°C for 30 min). The reactions were stopped by adding 1 M Na_2_CO_3_. Absorbances of the reaction supernatant were measured at 405 nm.

### 2.7. RNA isolation and qRT-PCR

*S. aureus* ATCC 6538 cells at OD_600_ of 0.05 were inoculated to 15 mL of LB medium in a 250 mL flat-bottomed flask and incubated for 6 h at 37°C with shaking at 250 rpm with or without 13-*cis*-retinoic acid (100 μg/mL). Cells were then treated with RNase inhibitor (RNAlater, Ambion, TX, USA) for preventing RNA degradation and harvested by centrifugation at 16,600 × g for 1 min. Total RNA was purified using an RNA isolation/purification kit (Qiagen RNeasy Mini Kit, Valencia, CA, USA), and additional step for cell lysis was performed using glass beads to enhance cell disruption. Briefly, acid-washed glass beads (Sigma-Aldrich, 150–212 μm, ~10 x vol. of cell pellet) were added in lysis buffer. The mixture was vortexed vigorously for 50 s and chilled down on ice between each vortex for 50 s, which was repeated twelve times. After breaking cells, supernatant was collected by centrifugation at 16,000 x g for 10 min and the rest of the procedure was followed by the manufacturer’s guidelines. qRT-PCR was applied to analyze the transcript levels of 32 biofilm- and toxin-related genes (*agrA, agrB, agrC, agrD, alsS, arlR, arlS, aur, clp9, coa, fibA, fibB, hla, icaA, icaR, isaA, lrgB, nuc1, nuc2, psmα, rbf, RNAIII, saeR, saeS, sarA, sarZ, seb, sigB, srrA, srrB, spa,* and *yycF*) in *S. aureus* ATCC 6538 cells. Primers used are listed in Supplementary Table S1, and *16s rRNA* was used as the housekeeping control. qRT-PCR was performed as previously described (Lee et al., 2022) using an SYBR™ Green qPCR Master Mix (Applied Biosystems, Foster City, United States) and an ABI StepOne Real-Time PCR System (Applied Biosystems). The changes of each gene expression were determined using two independent cultures and four reactions per gene.

### 2.8. Seed germination assay

*Brassica rapa* (Chinese cabbage) seeds were soaked in sterile H_2_O for 16 h, rinsed with water three times, sterilized by soaking in 95% ethanol first and then 3% sodium hypochlorite (both for 15 min) at 25°C, and rinsed with sterile H_2_O three times. Ten seeds per plate were carefully placed on Murashige and Skoog soft agar plates containing 0.7% agar and 0.86 g/L Murashige and Skoog (MS) and 13-*cis*-retinoic acid at 0, 20, 50, and 100 μg/mL, and then incubated at 25°C for 5 days. Seed germination rates and seedling lengths were then measured. Four independent cultures were used.

### 2.9. Chemical toxicity assay using a nematode model

*Caenorhabditis elegans fer-15(b26); fem-1(hc17)* strain was used to investigate the chemical toxicity of 13-*cis*-retinoic acid, as previously described (Kim et al., 2022b). Synchronized nematodes were carefully washed two times with M9 buffer (3 g/L KH_2_PO_4_, 6 g/L Na_2_HPO_4_, 5 g/L NaCl, 1 mM MgSO_4_). Approximately 40 worms were placed into the each well of 96-well plates containing M9 buffer (200 mL) and 13-*cis*-retinoic acid (50, 100, 200, or 500 μg/mL). Then, the plates were incubated for 10 days at 25°C. Four independent cultures were used. Survived nematode percentages was determined by responses to LED lights for 30 s and an iRiS™ Digital Cell Imaging System (Logos BioSystems).

### 2.10. Evaluation of absorption, distribution, metabolic, and excretion properties (ADME)

The drug-like properties of 13-*cis*-retinoic acid were evaluated using ADME software. The online web servers, *viz*, PreADMET^2^ Molinspiration^3^ and Gusar^4^ were accessed on March 15, 2023.

### 2.11. Statistical analysis

All results were analyzed by one-way ANOVA followed by Dunnett’s test in SPSS version 23 (SPSS Inc., Chicago, IL, United States). Results are presented as averages and standard deviations, and *p* values <0.05 are considered as a significant change.

## 3. Results

### 3.1. Antibiofilm activities of the three retinoic acids against *Staphylococcus aureus*

**Figure 1:**
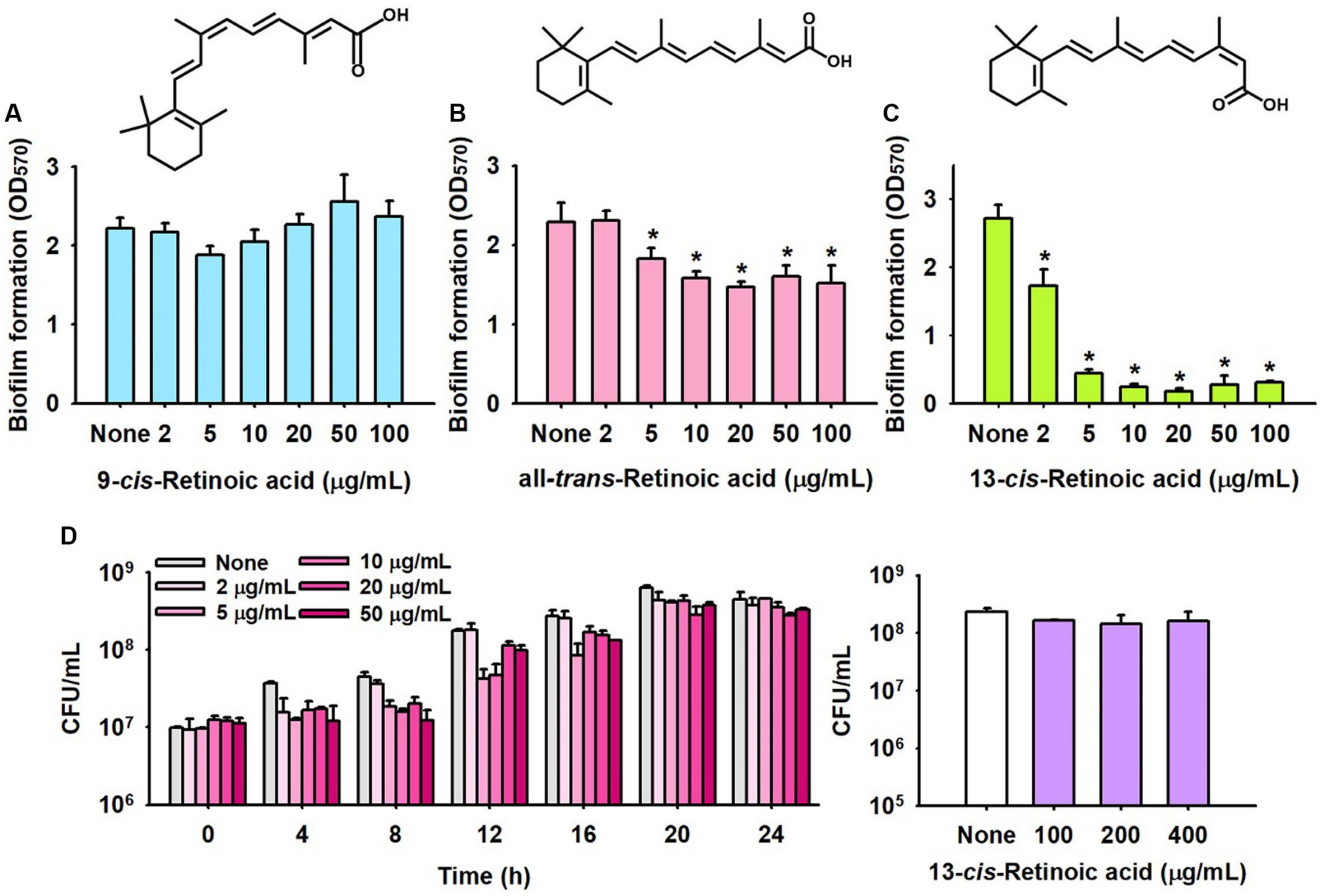
Effects of retinoic acids on biofilm formation and cell growth. Biofilm formation by *S. aureus* ATCC 6538 in the presence of 9-*cis*-retinoic acid **(A)**, all-*trans*-retinoic acid **(B)**, and 13-*cis*-retinoic acid **(C)**. Cell growth of *S. aureus* ATCC 6538 in the presence of 13-*cis*-retinoic acid **(D)** in 96-well polystyrene plates after culture for 24 h. ^*^p < 0.05 vs. non-treated controls (None).

The antibiofilm efficacies of three vitamin A_1_ metabolites, namely, all-*trans-*retinoic acid, 9-*cis*-retinoic acid, and 13-*cis*-retinoic acid, were initially tested at concentrations up to 100 μg/mL to investigate their effects on methicillin-sensitive *S. aureus* (MSSA 6538). Of these compounds, 13-*cis*-retinoic acid significantly inhibited biofilm formation, all-*trans*-retinoic acid had a weak inhibitory effect, but 9-*cis*-retinoic acid had no effect (Figures 1A–C). More specifically, 13-*cis*-retinoic acid at 10 μg/mL reduced *S. aureus* biofilm formation by 91%, while all-*trans*-retinoic acid at 100 μg/mL inhibited the biofilm formation by 34%. Also, the antimicrobial activity of 13-*cis*-retinoic acid was investigated by measuring colony-forming units, and at 50 μg/mL, it slightly delayed planktonic cell growth with a MIC of >400 μg/mL (Figure 1D). These results showed that the observed antibiofilm activity of 13-*cis*-retinoic acid was mainly due to its ability to inhibit biofilm formation rather than cell growth inhibition.

### 3.2. Antibiofilm efficacies of 13-*cis*-retinoic acid against other *Staphylococcus aureus* strains and other microbes

**Figure 2:**
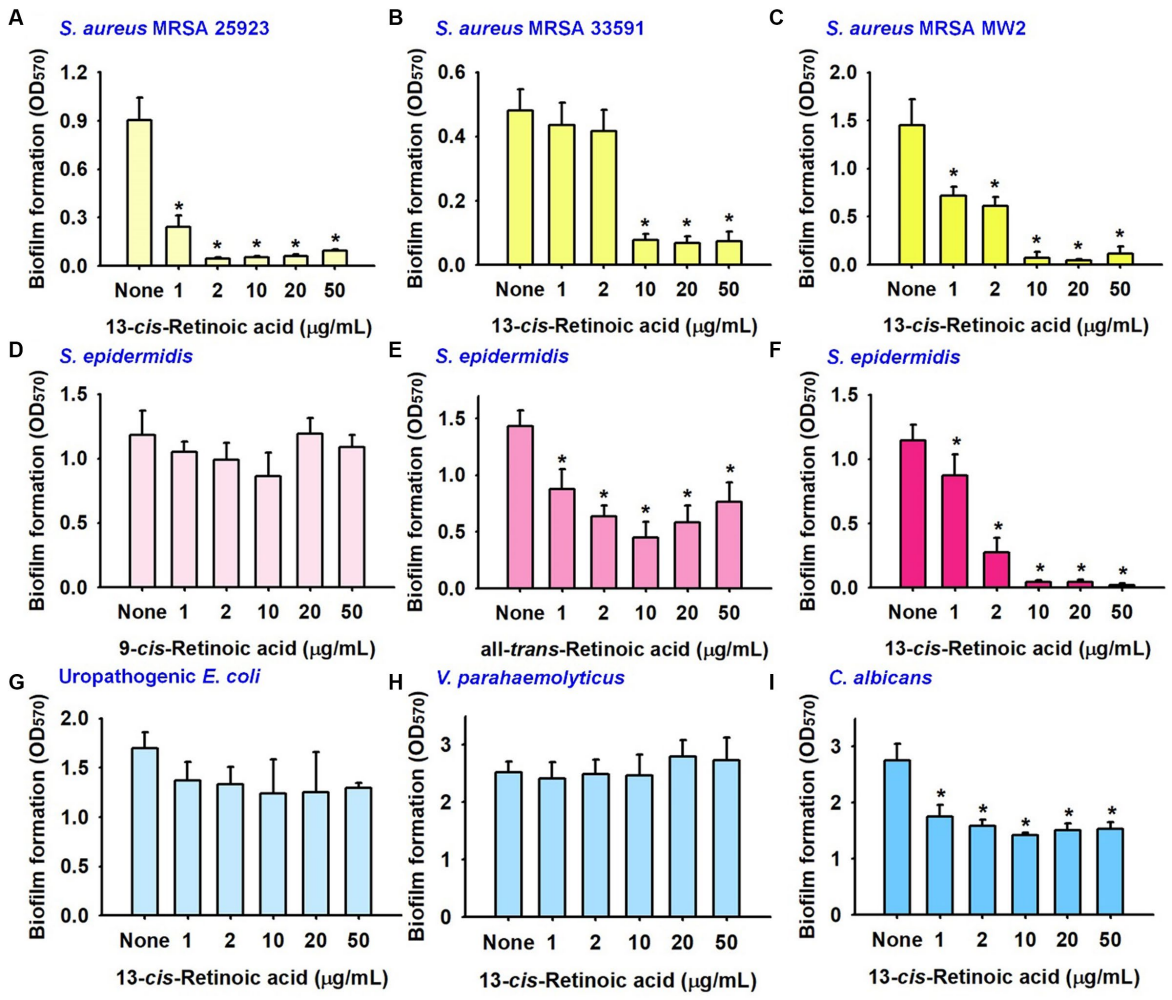
Inhibitory effects of retinoic acids on other biofilms. Biofilm formation by *S. aureus* ATCC 25923 **(A)**, MRSA 33951 **(B)**, MRSA MW2 **(C)**, *S. epidermidis* **(D–F)**, uropathogenic *E. coli* strain **(G)**, *V. parahaemolyticus* **(H)**, and *C. albicans* **(I)** in 96-well polystyrene plates after culture for 24 h. ^*^p < 0.05 vs. non-treated controls (None).

Further biofilm assays were performed on another MSSA 25923 strain and two methicillin-resistant *S. aureus* strains (MRSA 33591 and MW2). 13-*cis*-Retinoic acid potently reduced biofilm formation by MSSA 25923, MRSA 33591, and MRSA MW2 strains with MICs of >400 μg/mL (Figures 2A–C). Specifically, 13-*cis*-retinoic acid at 10 and 20 μg/mL decreased biofilm formation by MSSA 25923, MRSA 33591, and MRSA MW2 strains by ≥84%.

Also, the antibiofilm activities of three retinoic acids were investigated with a *S. epidermidis* strain. As was observed for *S. aureus*, *S. epidermidis* biofilms were strongly inhibited by 13-*cis*-retinoic acid, weakly inhibited by all-*trans*-retinoic acid, but unaffected by 9-*cis*-retinoic acid (Figures 2D–F).

The impact of 13-*cis*-retinoic acid on other biofilms was also investigated with a uropathogenic *Escherichia coli* strain, an aquatic pathogenic *Vibrio parahaemolyticus* strain, and a fungal *Candida albicans* strain. Unlike that observed for the five Staphylococcal biofilms, 13-*cis*-retinoic acid up to 50 μg/mL did not reduce biofilm formation by two Gram-negative pathogens (*E. coli* and *V. parahaemolyticus*) (Figures 2G,H) and only exhibited weak antibiofilm activity against *C. albicans* (Figure 2I). These results indicate that 13-*cis*-retinoic acid is active against Gram-positive Staphylococcal biofilms but not against Gram-negative bacteria.

### 3.3. Microscopic observations of the antibiofilm effects of retinoic acid on *Staphylococcus aureus*

**Figure 3:**
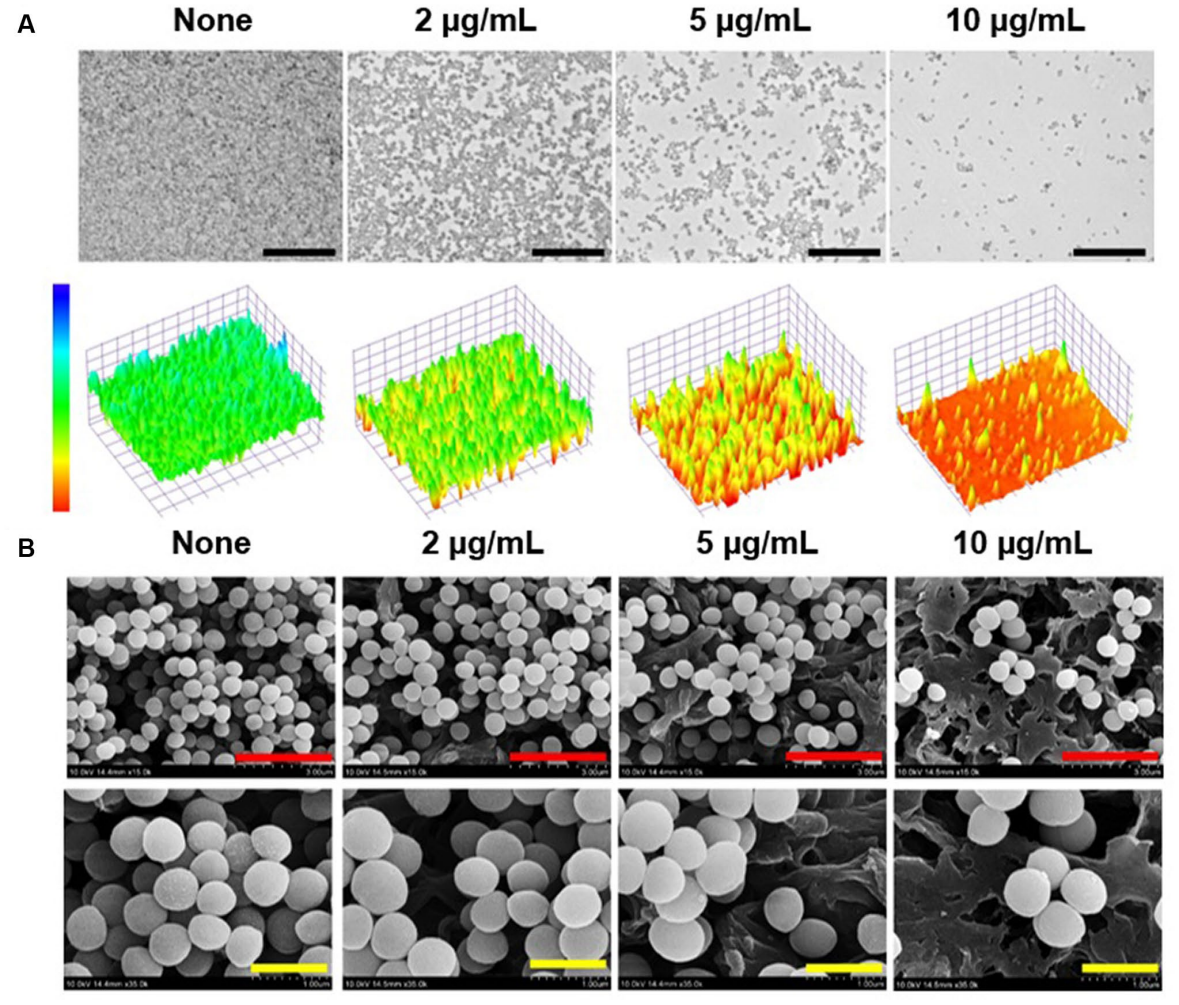
Antibiofilm effects of 13-*cis*-retinoic acid on *S. aureus*. Constructed color-coded 3D images of MSSA 6538 biofilms after culture for 24 h in the presence of 13-*cis*-retinoic acid **(A)**, and corresponding SEM images **(B)**. Black, red, and yellow scale bars represent 50, 3, and 1 μm, respectively.

Live imaging microscopy and SEM were utilized to observe biofilm reduction. For untreated biofilms, 3D color images obtained by bright-field microscopy were green, indicating dense biofilms, whereas 13-*cis*-retinoic acid at 2–10 μg/mL produced yellow to red colors, indicating weak to no biofilm formation (Figure 3A). SEM analysis also showed that 13-*cis*-retinoic acid markedly diminished the numbers of *S. aureus* cells in biofilms but did not affect *S. aureus* cell morphology (Figure 3B). These observations confirmed that 13-*cis*-retinoic acid at 2–10 μg/mL significantly inhibited *S. aureus* biofilm formation without affecting cell morphology.

### 3.4. Retinoic acids inhibited *Staphylococcus aureus* hemolytic activity and staphyloxanthin production

**Figure 4:**
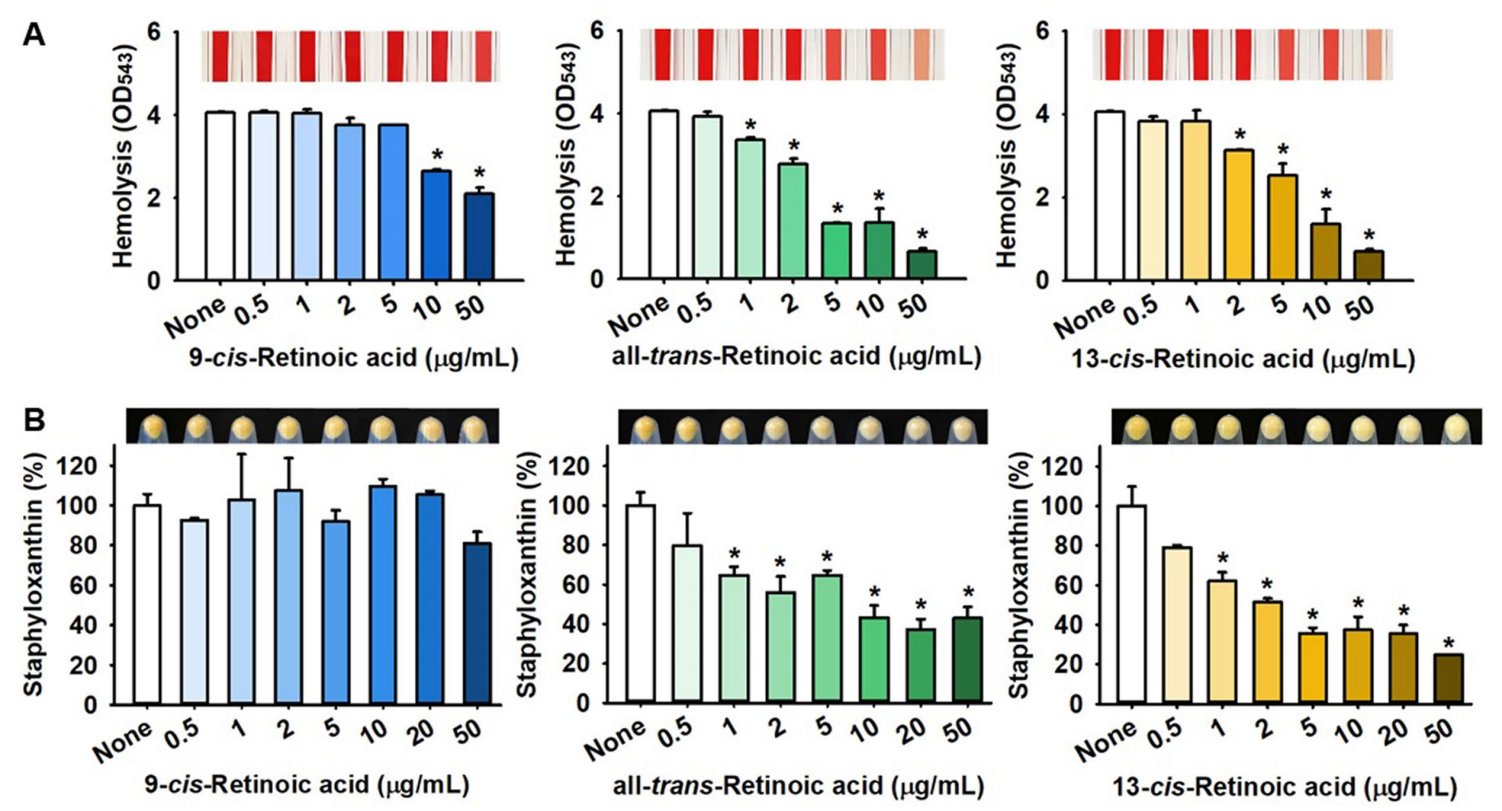
Effects of 13-*cis*-retinoic acid on hemolytic activity and staphyloxanthin production in *S. aureus*. Hemolysis **(A)** and staphyloxanthin production **(B)**. ^*^p < 0.05 vs. non-treated controls (None).

*S. aureus* is known to produce various virulence factors, including hemolysins, staphyloxanthin, and extracellular lipase, and thus, we investigated the effects of the three retinoic acids on their levels. Notably, all-*trans*-retinoic acid and 13-*cis*-retinoic acid dose-dependently reduced the red blood cell hemolytic activity of *S. aureus*, while 9-*cis-*retinoic acid showed only weak anti-hemolytic activity (Figure 4A). For example, all-*trans*-retinoic acid and 13-*cis*-retinoic acid at 50 μg/mL inhibited hemolytic activity by 84 and 83%, respectively, which partially reflected their antibiofilm activities (Figure 1).

Also, the effects of the three retinoic acids on yellow staphyloxanthin production in *S. aureus* were investigated. Interestingly, all-*trans*-retinoic acid and 13-*cis*-retinoic acid dose-dependently inhibited staphyloxanthin production, whereas 9-*cis-*retinoic acid did not, which paralleled our biofilm inhibition and hemolytic activity results (Figure 4B). However, 13-*cis*-retinoic acid did not affect extracellular lipase activity at concentrations of <50 μg/mL (Supplementary Figure S1).

### 3.5. Differential gene expression induced by 13-*cis*-retinoic acid in *Staphylococcus aureus*

**Figure 5:**
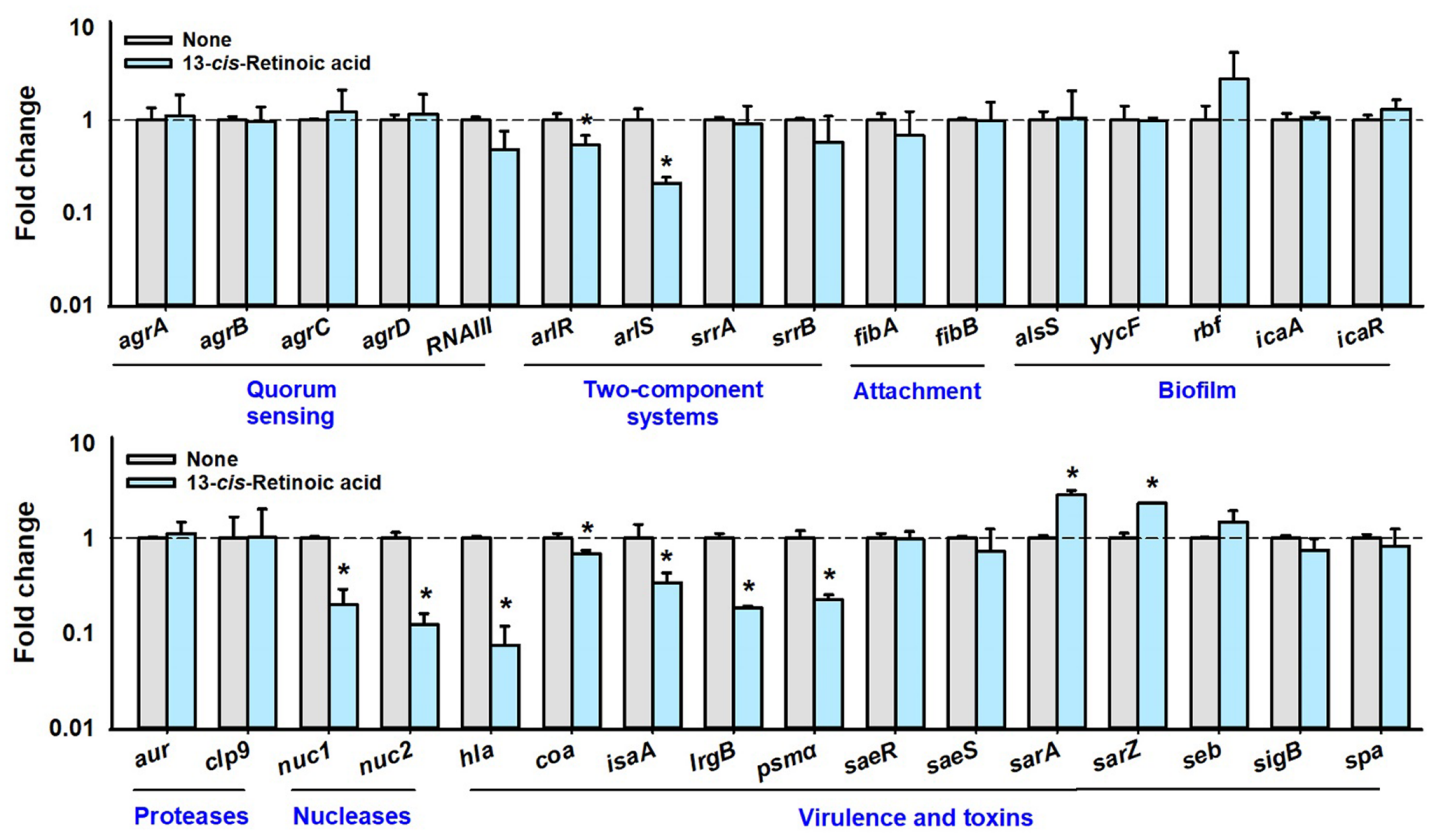
Effects of 13-*cis*-retinoic acid on gene expressions. Relative transcriptional profiles of biofilm- and virulence-related genes in *S. aureus* cells treated with 13-*cis*-retinoic acid at 100 μg/mL for 6 h with shaking at 250 rpm. Fold changes indicate transcriptional differences observed in treated vs. untreated (None) cells as determined by qRT-PCR. *16s rRNA* was used as the housekeeping gene. ^*^p < 0.05 vs. non-treated controls.

To investigate the molecular mechanisms responsible for the antibiofilm and antitoxin activities of 13-*cis*-retinoic acid on *S. aureus*, we used qRT-PCR to assess the expressions of 32 selected biofilm-, toxin-related genes and regulatory genes in *S. aureus* MSSA 6538 cells. 13-*cis*-Retinoic acid for 6 h incubation significantly downregulated the gene expression of the *arlRS* two-component system, α-hemolysin (*hla*), nuclease (*nuc1* and *nuc2*), coagulase *coaA*, staphylococcal antigen A *isaA*, antiholin-like protein *lrgB*, and *psmα* (phenol soluble modulins α) but slightly upregulated the expression of the transcriptional regulator *sarA* and *sarZ*. However, the expression of other genes tested was unchanged (Figure 5). Notably, 13-*cis*-retinoic acid suppressed *hla* expression 13-fold, which matches with its inhibitory effect on *S. aureus* hemolytic activity.

Further qRT-PCR has been conducted with a different growth condition of 10 h contact with 13-*cis*-retinoic acid (stationary growth phase) instead of 6 h contact (exponential growth phase). The changes of gene expression were attenuated since only the two-component kinase *arlS* was downregulated and other genes including QS-related genes were less affected after 10 h incubation (Supplementary Figure S2). The result indicates that gene expression is a dynamic process depending on incubation and growth stages.

### 3.6. Toxicity of 13-*cis*-retinoic acid in plant and nematode models

**Figure 6:**
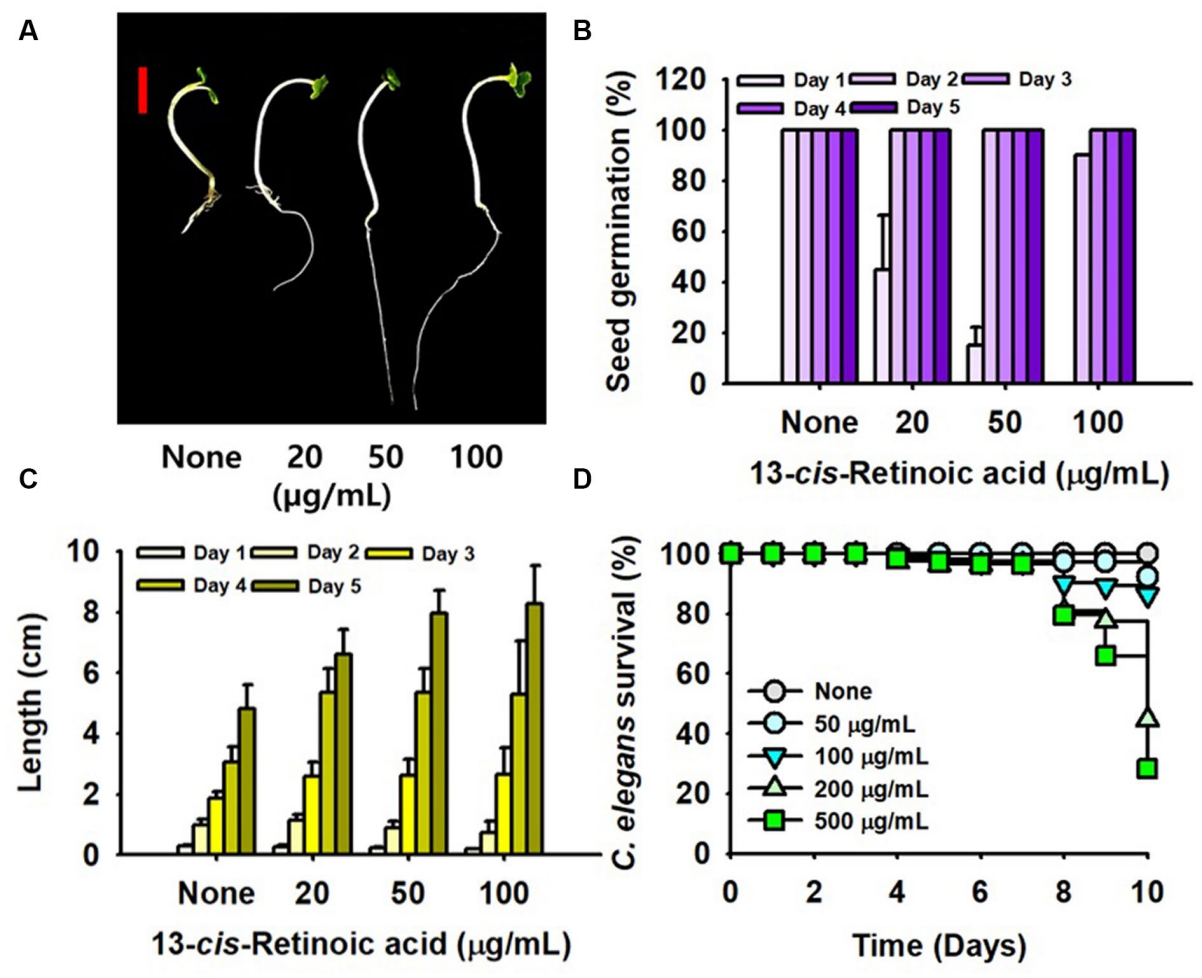
Toxicity of 13-*cis*-retinoic acid in the plant and nematode models. *B. rapa* seed growth **(A)**, germination rate **(B)**, and total length **(C)** cultured with or without different concentrations of 13-*cis*-retinoic acid at 25°C. **(D)** *C. elegans* survival was assessed in the presence or absence of 13-*cis*-retinoic acid for 10 days. The red scale bar in **(A)** represents 1 cm.

Chemical toxicity assessments of 13-*cis*-retinoic acid were performed using a *B. rapa* germination assay and a *C. elegans* model. Interestingly, 13-*cis*-retinoic acid dose-dependently increased (not decreased) plant root growth for 4 days (Figures 6A,C) and also slightly increased the seed germination rate (Figure 6B). In the nematode model, incubation with 13-*cis*-retinoic acid up to 50 μg/mL for 10 days was non-toxic (Figure 6D). While incubation for 8 days at concentrations of ≤500 μg/mL had no acute toxic effect, mild toxicity was observed for old nematodes after 8 days. These results suggest that 13-*cis*-retinoic acid may not be toxic to plants or nematodes in its antibiofilm concentration range (2–10 μg/mL).

### 3.7. ADME profiling of 13-*cis*-retinoic acid

*In silico* ADME profiling showed 13-*cis*-retinoic acid violated one (miLogP <5) of Lipinski’s Rule of Five but had acceptable human intestinal adsorption and skin barrier permeability and no fish toxicity. The ADME parameters investigated are detailed in Supplementary Table S2.

## 4. Discussion

This study demonstrates that retinoic acids, especially 13-*cis*-retinoic acid, inhibit biofilm formation of *S. aureus* and reduce its hemolytic activity and ability to produce staphyloxanthin without affecting its planktonic growth. Plant and nematode models and ADME analysis showed that 13-*cis*-retinoic acid is non-toxic at the active concentrations.

Retinoic acids are metabolites of all-*trans* retinol (vitamin A_1_), which is essential for the development of animals. All-*trans*-retinoic acid is the most abundant retinoic acid in nature, and its isomers, which include 9-*cis*-retinoic acid and 13-*cis*-retinoic acid, are present at markedly lower levels (Rühl et al., 2018). For example, the serum level of 13-*cis*-retinoic acid was the rage of 1.2–5.39 ng/mL in human (Li et al., 2019; Yang et al., 2020). The usage of 13-*cis*-retinoic acid was approved by the FDA in 1982 for the treatment of severe acne and has been shown to influence cellular differentiation, cell-cycle progression, cell survival, and apoptosis (Layton, 2009). Interestingly, 13-*cis*-retinoic acid was superior to all-*trans*-retinoic acid and 9-*cis*-retinoic acid for sebum suppression (Geiger et al., 1996).

It has been well established that acne vulgaris-associated inflammation may be exacerbated by *Cutibacterium acnes* and *S. aureus* (acne-associated bacteria) biofilm formation (Jahns et al., 2012; Tyner and Patel, 2016), which suggests the inhibitory effect of 13-*cis*-retinoic acid on *S. aureus* biofilm formation might be useful for treating acne. Therefore, we suggest studies be conducted to determine the impact of 13-*cis*-retinoic acid on anaerobic *C. acnes*.

Our transcriptomic study showed that 13-*cis*-retinoic acid repressed the expressions of regulatory *arlRS* genes and virulence factor genes (nuclease *nuc1* and *nuc2*, *psmα*, and α-hemolysin *hla*) in *S. aureus* (Figure 5). The ArlRS two-component system affects several cellular processes in *S. aureus*, including biofilm formation, autolysis, capsule synthesis and virulence (Crosby et al., 2020). Mutations in *arlRS* were reported to promote *S. aureus* biofilm formation (Toledo-Arana et al., 2005) and to inhibit adhesion to human endothelial cells and vascular structures (Kwiecinski et al., 2019). ArlRS has also been reported to be important for virulence in several animal infection models (Toledo-Arana et al., 2005). The nucleases Nuc1 and Nuc2 proteins are involved in biofilm structure and bacterial aggregation (Beenken et al., 2012; Yu et al., 2021), and phenol-soluble modulins (PSMs) are a family of toxins that act as key biofilm structuring factors in *S. aureus* (Periasamy et al., 2012). These previous studies support our findings that 13-*cis*-retinoic acid down-regulates these important biofilm regulators and thus inhibits biofilm formation.

**Figure 7:**
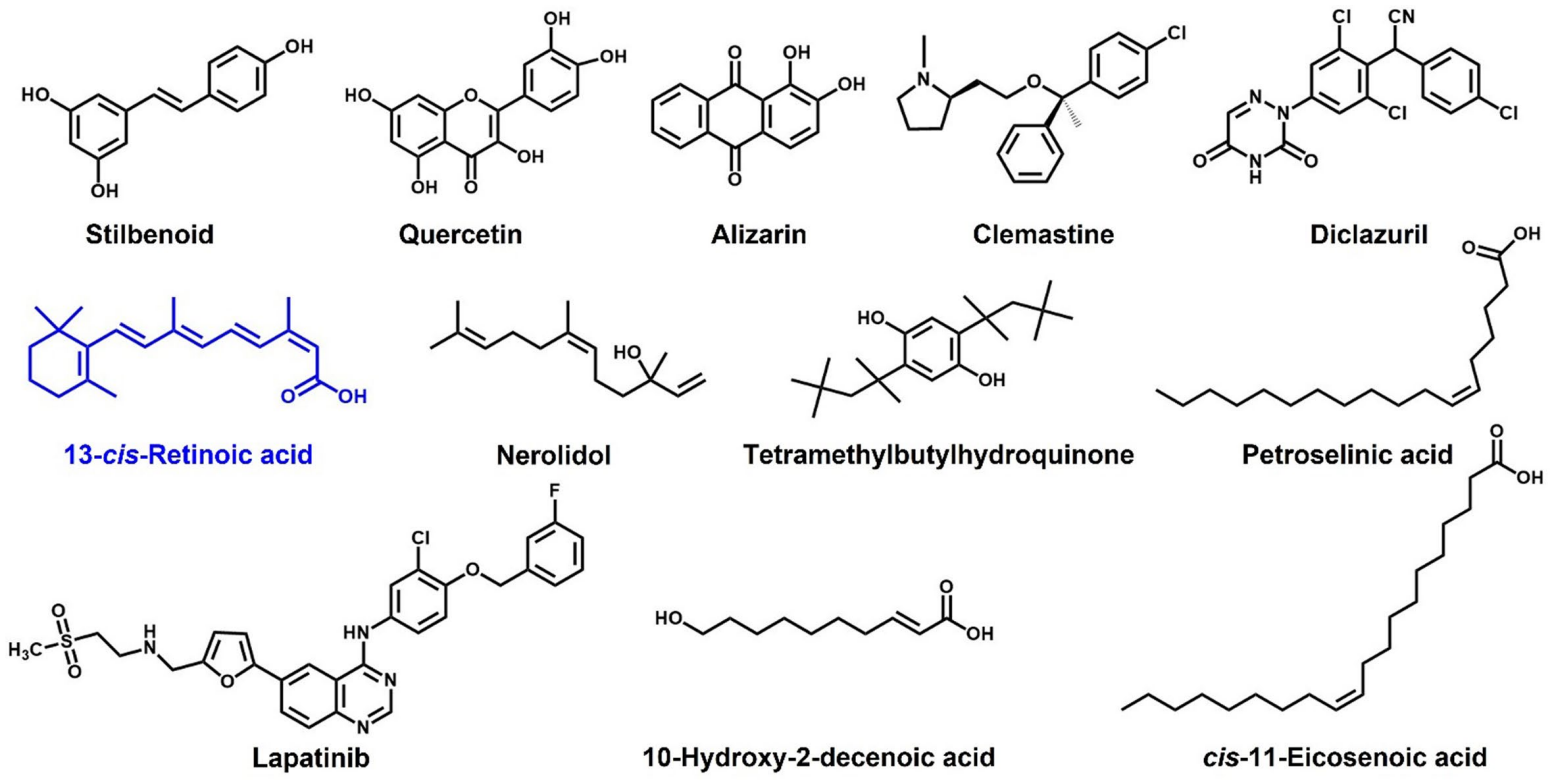
Structures of compounds reported to have antibiofilm and anti-hemolytic effects on *S. aureus.*

Our observations indicate that 13-*cis*-retinoic acid inhibits hemolytic activities (Figure 4) by suppressing the gene expression of α-hemolysin *hla* (Figure 5), a toxin that plays an important role in the pathogenesis of *S. aureus* infections by causing hemolysis (Divyakolu et al., 2019) and positively regulating *S. aureus* biofilm formation (Caiazza and O'Toole, 2003). Previous studies have shown that stilbenoid (Lee et al., 2014b), several flavonoids (Cho et al., 2015), alizarin (Lee et al., 2016), clemastine (Shang et al., 2022), diclazuril (Zheng et al., 2021), tetramethylbutylhydroquinone (Kim et al., 2022), nerolidol (Lee et al., 2014a), 10-hydroxy-2-decenoic acid (Gao et al., 2022), petroselinic acid (Lee et al., 2022), *cis*-11-eicosenoic acid (Lee et al., 2017), and lapatinib (Liu et al., 2022) have antibiofilm and anti-hemolytic effects on *S. aureus*. These findings suggest a positive relationship exists between antibiofilm and anti-hemolysis activities. Interestingly, structural comparisons of these compounds indicate that a hydroxyl or acid group and an alkyl chain with that of retinoic acid positively influence antibiofilm and anti-hemolysis activities (Figure 7). We suggest molecular docking studies be conducted on Hla protein and these compounds to identify some possible targets in Hla.

Notably, 13-*cis*-retinoic acid did not affect the expressions of *agr* or *RNAIII* quorum sensing system (Figure 5), which is somewhat intriguing as the Agr system contributes to *S. aureus* biofilm formation and biofilm dispersal (Boles and Horswill, 2011), and RNAIII is a key effector of Agr system that binds to AgrA and positively regulates hemolysins (Koenig et al., 2004). Thus, our transcriptomic results (Figure 5) indicate that the inhibitions of biofilm formation and hemolysis by 13-*cis*-retinoic acid are less associated with the Agr and RNAIII systems.

It has been previously shown that oral administration of 13-*cis*-retinoic acid has no direct antimicrobial effect (Layton, 2009), which concurs with our results (Figure 1D). Oral 13-*cis*-retinoic acid has been reported to be an effective acne treatment but may induce mood changes and mucocutaneous problems (Layton, 2009). However, our toxicity (Figure 6) and ADME results (Supplementary Table S2) suggest that 13-*cis*-retinoic acid is environmentally non-toxic and displays acceptable skin permeability, which suggests oral or dermal administration might be a feasible way of treating biofilm-associated *S. aureus* infections.

## Data availability statement

The original contributions presented in the study are included in the article/Supplementary material, further inquiries can be directed to the corresponding author.

## Ethics statement

The manuscript presents research on animals that do not require ethical approval for their study.

## Author contributions

JM, YT, and JL: conceptualization. IP and J-HL: methodology, software, validation, formal analysis, investigation, data curation, and visualization. JM and JL: resources. JL: writing of the manuscript and project administration. All authors contributed to the article and approved the submitted version.

## Funding

This study was supported by grants from the Basic Science Research Program of the National Research Foundation of Korea (NRF) funded by the Ministry of Education (2021R1I1A3A04037486), the NRF funded by the Korean government (MSIT) (2021R1A2C1008368), and by the Priority Research Center Program of the NRF funded by the Ministry of Education (2014R1A6A1031189).

## Conflict of interest

The authors declare that the research was conducted in the absence of any commercial or financial relationships that could be construed as a potential conflict of interest.

## Publisher’s note

All claims expressed in this article are solely those of the authors and do not necessarily represent those of their affiliated organizations, or those of the publisher, the editors and the reviewers. Any product that may be evaluated in this article, or claim that may be made by its manufacturer, is not guaranteed or endorsed by the publisher.

## Footnotes

^1^https://imagej.nih.gov/ij/index.html

^2^https://preadmet.qsarhub.com/

^3^https://www.molinspiration.com/

^4^https://www.way2drug.com/gusar/
