# Supplementary material for: Antivirulence activities of retinoic acids against Staphylococcus aureus
Source: Front Microbiol. 2023 Sep 13;14:1224085. doi: 10.3389/fmicb.2023.1224085 (PMC10525321; doi:10.3389/fmicb.2023.1224085)
Supplement: Supplementary file 1 [file Data_Sheet_1.docx]

Supplementary Material

**Antivirulence activities of retinoic acids against *Staphylococcus aureus***

Inji Park^1†^, Jin-Hyung Lee^1†^, Jin Yeul Ma^2^, Yulong Tan^3^, and Jintae Lee^1*^

*** Correspondence:** Jintae Lee, [jtlee@ynu.ac.kr](mailto:jtlee@ynu.ac.kr)

**Supplementary Table 1.** Sequences of the primers used for quantitative RT-PCR.

| Gene | Name | Primer |
| --- | --- | --- |
| *16S*  *rRNA* | A component of ribosomes | Forward 5'-TGT TTG ACG ATG TTT GAG CA-3' |
|  |  | Reverse 5'-CCT TCC TCC AGT TCA GAT GC -3' |
| *agrA* | Quorum-sensing regulator A | Forward 5'-TGA TAA TCC TTA TGA GGT GCT T-3' |
|  |  | Reverse 5'-CAC TGT GAC TCG TAA CGA AAA-3' |
| *agrB* | Quorum-sensing regulator B | Forward 5'- TGC AAA GTC TTC GAT ACT TTG TTA CA -3'  Reverse 5'- GGT ATA GGT TGC TTT TTC GTT GCT-3' |
| *agrC* | Quorum-sensing regulator C | Forward 5'- AAG ATG ACA TGC CTG GCC TA -3'  Reverse 5'- TGT GCA CGT AAA ATT TTC GCA G -3' |
| *agrD* | Quorum-sensing regulator D | Forward 5'- CAT TCC TGT GCG ACT TAT TAA ACG -3'  Reverse 5'- CGT GTA ATT GTG TAA ATT CTT TTG C -3' |
| *alsS* | Acetolactate synthase | Forward 5'- AAT GGT ATG CAA ACG CTT GGT -3'  Reverse 5'- GCG TAT TAG GGC GCA CAA GT -3' |
| *arlR* | Response regulator | Forward 5'-TTA CGG TGC AGG CGA TTA TAT AG-3' |
|  |  | Reverse 5'-TAC CGT TGA CAT CGA TAA TAT CC-3' |
| *arlS* | Histidine-protein kinase | Forward 5'-TGG AAT ACC AAT TCC ATG ATC T-3' |
|  |  | Reverse 5'-TGC AAT CAA ATA TGA TGT GAA GAA-3' |
| *aur* | Zinc metalloproteinase aureolysin | Forward 5'-ACC GTG TGT TAA TTC GTG TGC TA-3' |
|  |  | Reverse 5'-ATG GTC GCA CAT TCA CAA GTT T-3' |
| *clp9* | Serine protease | Forward 5'-CAG GTA CCA TCA CTT CAT C-3' |
|  |  | Reverse 5'-GGT TCA CAA ATT GAT GAC AAC G-3' |
| *coa* | Coagulase | Forward 5'-CAC GGA AAT GGC CAA GTA TC-3' |
|  |  | Reverse 5'-TCG GAC GAG CTC CAT ATG AT-3' |
| *fibA* | Fibronectin-binding protein A | Forward 5'- GAT ACA AAC CCA GGT GGT G -3'  Reverse 5'- TGT GCT TGA CCA TGC TCT TC -3' |
| *fibB* | Fibronectin-binding protein B | Forward 5'- TGT GCT TGA CCA TGC TCT TC -3'  Reverse 5'- AGT TGA TGT CGC GCT GTA TG -3' |
| *hla* | α-Hemolysin | Forward 5'-CGG CAC ATT TGC ACC AAT AAG GC-3' |
|  |  | Reverse 5'-GGT TTA GCC TGG CCT TCA GC-3' |
| *icaA* | Intercellular adhesion A | Forward 5’-TGA ACC GCT TGC CAT GTG-3’ |
|  |  | Reverse 5’-CAC GCG TTG CTT CCA AAG A-3’ |
| *icaR* | Intercellular locus regulator | Forward 5'-TCG AAC TAT TCA ATT GAT GCT TTA-3' |
|  |  | Reverse 5'-CAG AAA ATT CCT CAG GCG TA-3' |
| *isaA* | Transglycosylase | Forward 5'-GCT CAA ATC ATG GCT CAA CGT-3' |
|  |  | Reverse 5'-TTG ATT CAC GAG CGA TGA TTG-3' |
| *lrgB* | Anti-hollin-like protein | Forward 5'-TCG GAG GTA TTG GTA TCG-3' |
|  |  | Reverse 5'-CTG CTT GAG GTA ACA TTG A-3' |
| *nuc1* | Nuclease | Forward 5'-CAC CTG AAA CAA AGC ATC CTA A-3' |
|  |  | Reverse 5'-TAT ACG CTA AGC CAC GTC CAT-3' |
| *nuc2* | Nuclease | Forward 5'-ATG GAC GTG GCT TAG CGT AT-3' |
|  |  | Reverse 5'-TGA CCT GAA TCA GCG TTG TC-3' |
| *psmα* | Phenol soluble modulins α | Forward 5'-ACC CAT GTG AAA GAC CTC CTT TGT-3' |
|  |  | Reverse 5'-ATG GGT ATC ATC GCT GGC ATC-3' |
| *rbf* | Regulator of biofilm formation | Forward 5'-TTA GAA GGA ATC TTT AAA ACC TTA TTG AAT AA-3' |
|  |  | Reverse 5'-TTG TGA ATT TTT CTT CTT CGG ACA-3' |
| *RNAⅢ* | Transcriptional regulator | Forward 5'-ATC GAC ACA GTG AAC AAA TTC AC-3' |
|  |  | Reverse 5'-CTC TAC TAG CAA ATG TTA CTC AC-3' |
| *saeR* | Response regulator | Forward 5'-GCC TTA ACT TTA GGT GCA GAT GAC TAT GTC-3' |
|  |  | Reverse 5'-CGA CAG TTG TTC AAC TGG TTG ATG ATG G-3' |
| *saeS* | Membrane-associated protein kinase | Forward 5'- CGT ACA TTC AGA GTA GAA AAC TCT CGT AAT AC -3'  Reverse 5'- GTT GCG CGA GTT CAT TAG CTA TAT AT -3' |
| *sarA* | Transcriptional regulator | Forward 5'-GAG TTG TTA TCA ATG GTC-3' |
|  |  | Reverse 5'-GTT TGC TTC AGT GAT TCG-3' |
| *sarZ* | HTH-type transcriptional regulator | Forward 5'-CCT ATA CTG GTT ACA TTG TTT TAA TGG -3'  Forward 5'-TGG TGT CAG TGT TCC AGA ATC -3' |
| *seb* | Enterotoxin B | Forward 5'-TGT TCG GGT ATT TGA AGA TGG -3'  Reverse 5'-CGT TTC ATA AGG CGA GTT GTT-3' |
| *sigB* | RNA Polymerase sigma factor | Forward 5'-AAG TGA TTC GTA AGG ACG TCT-3' |
|  |  | Reverse 5'-TCG ATA ACT ATA ACC AAA GCC T-3' |
| *srrA* | Transcriptional regulator (two-component regulatory system) | Forward 5’- TAA TGT TGC CTG AAA TGG ATG G -3’  Reverse 5’- CAA CAC GGT TTG TTT CTT CAC CT -3’ |
| *srrB* | Sensor protein (two-component regulatory system) | Forward 5'- AGC CGG CTA AAT AGT GTC GT -3'  Reverse 5'- ATG GCA TTT TCG GTT TCT TG -3' |
| *spa* | Protein A | Forward 5'-ACC AGA AAC TGG TGA AGA AAA TCC-3' |
|  |  | Reverse 5'-TAA CGC TGC ACC TAA GGC TAA TG-3' |
| *yycF* | Transcriptional regulator | Forward 5'- TGG CGA AAG AAG ACA TCA -3'  Reverse 5'- AAC CCG TTA CAA ATC CTG -3' |

**Supplementary Table 2.** ADME (absorption, distribution, metabolism, and excretion) profiles of 13-*cis*-retinoic acid.

| Property | 13-*cis*-Retinoic acid |
| --- | --- |
| Lipinski rule of five | Suitable |
| Lipinski rule of five violations | 1 |
| Plasma protein binding | 100% |
| Blood brain barrier permeability | 3.88534 |
| Skin permeability | -0.67673 |
| Human intestinal absorption | 97.94% |
| Caco 2 | 22.8974 |
| Mouse carcinogenicity | Positive |
| Rat carcinogenicity | Positive |
| Acute algae toxicity | 0.00506299 |
| Acute fish toxicity (medaka) | 0.00143016 |
| Acute fish toxicity (minnow) | 0.000491265 |
| In vitro hERG inhibition | Medium risk |
| miLogP | 5.80 |
| Mol volume | 315.59 |
| TPSA | 37.30 |
| GPCR ligand | 0 |
| Ion channel modulator | 0.14 |
| Kinase inhibitor | -0.18 |
| Nuclear receptor ligand | 1.13 |
| Protease inhibitor | -0.03 |
| Enzyme inhibitor | 0.57 |
| Rat IP LD50 classification | Class 5 in AD |
| Rat IV LD50 classification | Class 4 in AD |
| Rat oral LD50 classification | Class 5 in AD |
| Rat SC LD50 classification | Class 4 in AD |


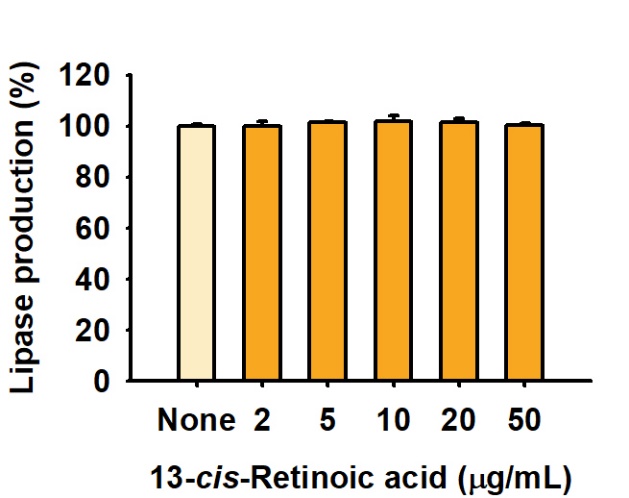


**Supplementary Figure 1.** Effects of 13-*cis*-retinoic acid on extracellular lipase production in *S. aureus* ATCC 6538.


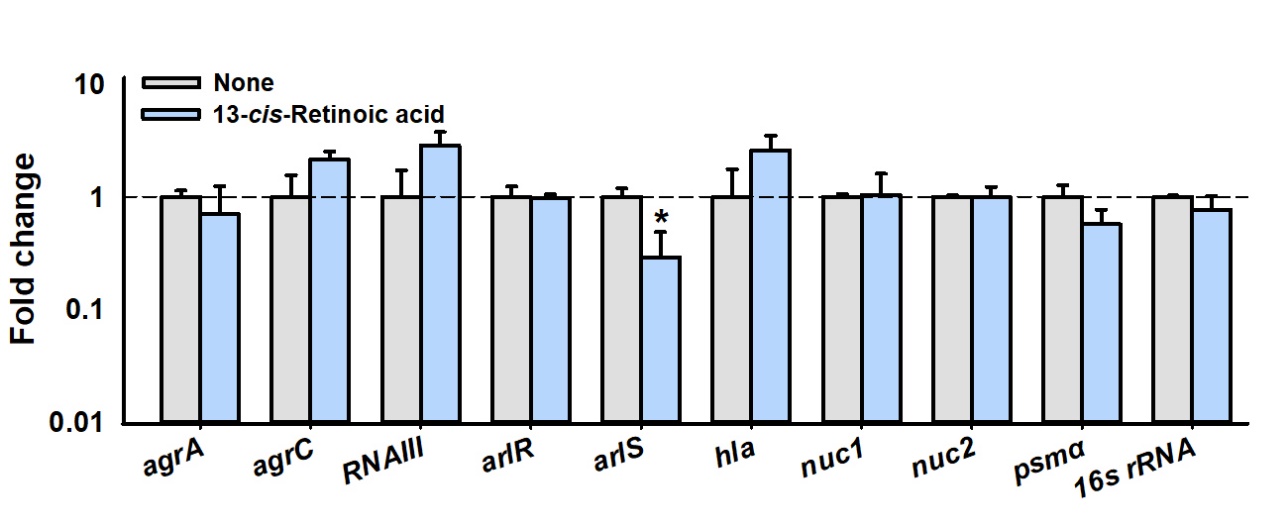


**Supplementary Figure 2.** Relative transcriptional profiles of biofilm- and virulence-related genes in *S. aureus* cells treated with 13-*cis*-retinoic acid at 100 μg/mL for 10 h with shaking at 250 rpm. Fold changes indicate transcriptional differences observed in treated *vs*. untreated (None) cells as determined by qRT-PCR. *16s rRNA* was used as the housekeeping gene. * *p* < 0.05 *vs.* non-treated controls.
